# Repositioning approved drugs for the treatment of problematic cancers using a screening approach

**DOI:** 10.1371/journal.pone.0171052

**Published:** 2017-02-06

**Authors:** Hristo P. Varbanov, Fabien Kuttler, Damiano Banfi, Gerardo Turcatti, Paul J. Dyson

**Affiliations:** 1 Institut des Sciences et Ingénierie Chimiques, Ecole Polytechnique Fédérale de Lausanne (EPFL), Lausanne, Switzerland; 2 Biomolecular Screening Facility, Ecole Polytechnique Fédérale de Lausanne (EPFL), Lausanne, Switzerland; University of South Alabama Mitchell Cancer Institute, UNITED STATES

## Abstract

Advances in treatment strategies together with an earlier diagnosis have considerably increased the average survival of cancer patients over the last four decades. Nevertheless, despite the growing number of new antineoplastic agents introduced each year, there is still no adequate therapy for problematic malignancies such as pancreatic, lung and stomach cancers. Consequently, it is important to ensure that existing drugs used to treat other types of cancers, and potentially other diseases, are not overlooked when searching for new chemotherapy regimens for these problematic cancer types. We describe a screening approach that identifies chemotherapeutics for the treatment of lung and pancreatic cancers, based on drugs already approved for other applications. Initially, the 1280 chemically and pharmacologically diverse compounds from the Prestwick Chemical Library® (PCL) were screened against A549 (lung cancer) and PANC-1 (pancreatic carcinoma) cells using the PrestoBlue fluorescent-based cell viability assay. More than 100 compounds from the PCL were identified as hits in one or both cell lines (80 of them, being drugs used to treat diseases other than cancer). Selected PCL hits were further evaluated in a dose-response manner. Promising candidates for repositioning emanating from this study include antiparasitics, cardiac glycosides, as well as the anticancer drugs vorinostat and topotecan.

## Introduction

Cancer is amongst the world´s leading causes of death, with the greatest economic impact from premature death and disability from all causes of death worldwide [1,2]. The term cancer is given to a collection of diseases characterized by the uncontrolled growth and spread of abnormal cells, and includes hundreds of subtypes that differ dramatically in incidence and prognosis [3,4]. Over the last few decades improvements in treatment strategies, together with an earlier diagnosis, have significantly increased the average survival of cancer patients [5,6]. Nevertheless, there is a wide variation in response rates between cancer types, with, for example, the predicted ten-year net survival for patients diagnosed during 2010–2011 in the UK ranging from 98% for testicular cancer to just 1% for pancreatic cancer (S1 Fig) [6,7]. Despite the increasing number of new anticancer drugs introduced each year [8], no adequate therapy exists for problematic malignancies such as pancreatic, lung, brain and stomach cancers. Consequently, it is important to ensure that existing drugs used to treat other types of cancers, and potentially other diseases, are not overlooked when searching for new chemotherapy regimens for these problematic cancer types.

Drug discovery and development frequently employs high-throughput screening (HTS) of libraries that may contain several hundreds of thousands of compounds typically covering the commercially available chemical diversity. HTS has had a low success rate in discovering drugs *de novo* as primary hits often have poor bioavailability or toxicity profiles [9]. As a result, recent HTS approaches include the use of approved (marketed) drugs libraries to identify new biological activities that could lead to repositioning of the compound (also referred as drug repurposing) [10]. Examples include the selective optimization of side activities (SOSA) approach [11] where a set of approximately 1000 structurally and therapeutically diverse, well-characterized drug molecules is screened for activity towards new pharmacological targets. Subsequently, the hits are rationally modified to amplify the new activity and reduce or eliminate other pharmacological effects, transforming the newly observed ‘side activity’ into the main effect. An advantage of this approach is that all identified hits are compounds with confirmed safety and bioavailability in humans, potentially leading to lower costs with subsequent clinical development, shorter approval times and higher approval rates [9,10]. Compound libraries have also been screened against comparatively complex 3D cell culture systems that mimic the tumor microenvironment [12,13].

A number of chemotherapy regimens are available for treatment of lung cancer [14,15], however, it is among the top five cancers with the lowest 5-year survival rate [4,16]. Pancreatic cancer is one of the most deadly malignancies with a 5-years survival rate of only 2.4% [4,7,17]. Surgical resection offers the only potentially curative treatment, but in the majority of the cases the disease is inoperable at the time of diagnosis and chemotherapy remains the standard-of-care in most countries [15,17]. Consequently, we used a cell based HTS assay to identify new potential chemotherapeutics for the treatment of problematic cancers, i.e. lung and pancreatic, based on drugs already approved for other applications. In this article we disclose the outcome of these screening studies and show that there are several promising drugs approved to treat other diseases that should be considered for repositioning.

## Results and discussion

### Screening assays

The 1280 chemically and pharmacologically diverse compounds (ca. 90% being FDA-approved drugs) from the Prestwick Chemical Library (PCL) [18] were screened against chemoresistant non-small lung cancer (A549) and pancreatic carcinoma (PANC-1) cell lines. Experiments were conducted in 384-well format in reverse mode (PCL compounds were dispensed on the plates before seeding of the cells) due to the high amount of compounds to be screened in multiple conditions and the semi-automation required. The anthracycline cytotoxic antibiotic doxorubicin hydrochloride (DOX, S2 Fig) was used as a representative positive control from the PCL as it exhibits a broad spectrum of activity. DOX is a cell-cycle non-specific chemotherapeutic agent with multiple reported mechanisms of action and it is used in various combination chemotherapy regimens for the treatment of a wide range of cancers [15,16,19,20]. Moreover, its cytotoxic effects are usually exhibited at relatively low concentrations, i.e. IC_50_ values are typically in the low micromolar to nanomolar range [21,22]. The screening window coefficient (Z’-factor) was used as an indicator for assay development and optimization (i.e. setting of cell number, exposure time, volumes used, etc.) and as a statistical tool for the assay quality assessment [23]. The quality of the assay was considered sufficient for screening and automation when Z’ >0.5.

Short incubation times (e.g. 24 h) tend to be preferable for cell-based HTS assays, however, due to the slow growth rate of the A549 and PANC-1 cell lines with population doubling times (PDT) of ≥20 h and their high resistance to known chemotherapeutics, the assays were optimized with incubation times of 50 h for the A549 cells and 72 h for the PANC-1 cells. The Presto Blue fluorescent-based cell viability assay was selected as readout for the screens due to its simplicity, sensitivity, high experimental efficiency and performance [24]. It represents a valuable, HTS-compatible alternative to the widely used MTT colorimetric assay [25].

**Fig 1:**
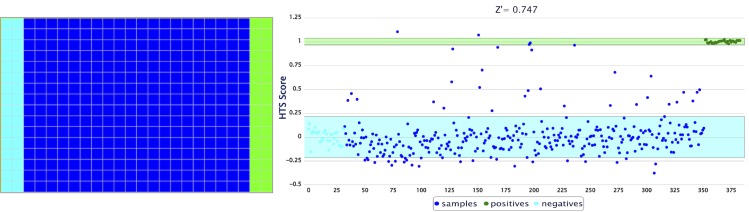
Schematic of the HTS assay representing a 384-well screening plate (left) and the respective data evaluation and visualization after normalization (right). First two columns (pale blue): negative control (0.1% DMSO; no drug added); Last two columns (green): positive control (DOX, 10 μM, final DMSO concentration 0.1%); Middle columns (dark blue) test compounds (PCL drugs, 10 μM, final DMSO concentration 0.1%, 1 well/compound, 320 compounds/plate). The full PCL can be processed on four plates. Cell suspension (A549 cells shown here) is added to every well. The Z’-factor for plate validation is calculated using the fluorescence signals of the control wells (the green and blue shaded areas on the right side denote the average ± 3 x SD of the negative and the positive controls, respectively). In this example a Z’-factor of 0.747 is suitable to validate the plate.

The PCL was tested initially at a fixed concentration of 10 μM with DOX taken as a positive control and an equivalent volume of DMSO was used as negative control (see Fig 1 for the plate layout). The results from the screens were normalized to the controls for every plate and presented as HTS scores, where a score of 0 corresponds to the average fluorescence intensity of the negative control wells (and denotes no cytotoxic activity) and a score of 1 to that of the positive control wells, and indicates very active compounds (see Fig 1 for an example of the screening outcome from a 384-well plate). Hit compounds (or PCL hits) were identified and statistically validated when their HTS scores are higher than the average of the negative controls + 3 x SD. The primary scores for the PCL hits are based on the mean from three independent experiments, each performed in duplicate (averaged HTS scores are listed in S1 and S2 Tables).

The hit compounds found during the primary screen were subjected to an additional (confirmation) screen, employing the same experimental protocol used for the primary screen, where primary hits with score >0.7 were re-tested at a lower concentration (0.83 μM, instead of 10 μM).

### Comparative analysis and classification of hit compounds

**Fig 2:**
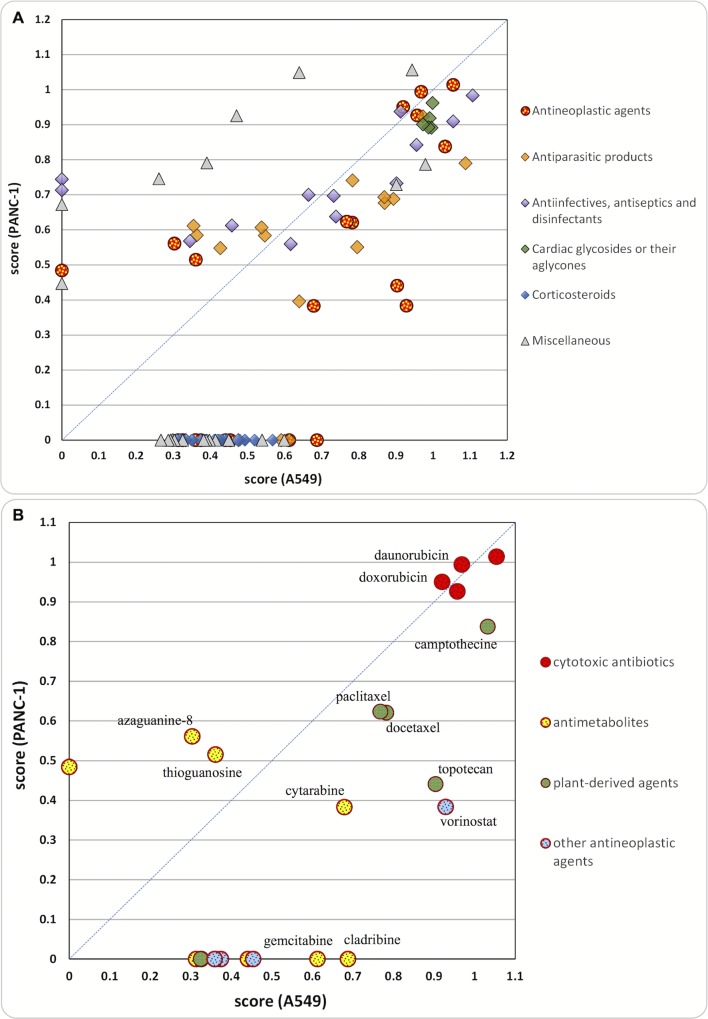
PCL compounds identified as hits in A549 and/or PANC-1 cells, plot based on their mean scores from the primary screens. A) all PCL hits; B) only hits which are used as antineoplastic drugs. Hits identified in only one of the cell lines are plotted and their score on the other is taken for zero. For all the names of the hit compounds, score values and pharmacological classification see S1 and S2 Tables.

Most of the identified PCL hits showed similar potency in both cancer cell lines, appearing around the mid-line of Fig 2. Nevertheless, somewhat fewer hits were found for the PANC-1 cells (52 hits, versus 98 hits for A549 cells), presumably as it is a more chemoresistant cancer cell line [15,17,21]. In addition, less active hits (with HTS scores <0.4) could not be detected in PANC-1 cells due to the narrower separation bands of the assay for this cell line.

#### Anticancer drugs

One of the most active class of compounds identified during the screens are cytotoxic antibiotics, including the positive control DOX, with mean scores >0.9 in both cell lines (Fig 2). Re-testing these compounds at a 10-times lower concentration during the confirmation assay resulted in decreased scores (0.45–0.7). Like doxorubicin, daunorubicin is an anthracycline and this group of compounds rank among the most effective anticancer drugs with activity against a wide range of tumors [15,19]. Both compounds have been trialed in various anticancer combination therapies [26,27] and doxorubicin is included in some non-platinum based regimens for treatment of lung cancer [14,16]. Doxorubicin and daunorubicin were tested in a dose-response manner resulting in IC_50_ values in the range 0.3–0.5 μM in both cell lines (see below), demonstrating the potential of these compounds as chemotherapeutics for the treatment of these cancers.

Several anticancer drugs from the group of the antimetabolites, i.e. pyrimidine and purine analogues, were identified as hits against the A549 cells during the screening with HTS scores ranging from 0.3 to 0.7. Subsequent dose-response studies revealed IC_50_ values in the submicromolar range for cladribine, cytarabine and gemcitabine. Antimetabolites are S-phase specific chemotherapeutic agents, which interfere with key steps in cellular metabolism, e.g. DNA and RNA synthesis, and show activity against various malignancies [16]. Gemcitabine is a pyrimidine antimetabolite used as a single agent and in combination chemotherapy, most frequently with platinum drugs, for the treatment of both lung and pancreatic cancers [14]. However, as with most of the pyrimidine antimetabolites identified as hits in the A549 screens, gemcitabine did not appear as hit in the PANC-1 screens.

Other anticancer drugs identified as hits during the screens include plant-derived agents, i.e. taxanes and camptothecines, protein kinase inhibitors (only in A549 cells) and vorinostat. The plant alkaloids paclitaxel and docetaxel displayed HTS scores of ca. 0.7, which were retained also in the low concentration setting of the confirmation screen. Both compounds demonstrated cytotoxic effects at very low concentration with IC_50_ <0.05 μM (see below) in accordance with literature data [22,28]. Paclitaxel and docetaxel are both in the taxane family of antineoplastic drugs, which act as mitotic inhibitors (targeting the microtubules) and are applied in the chemotherapy of various cancers, including NSCLC and SCLC, [14] and are undergoing clinical trials for treatment of pancreatic cancer [17]. Other mitotic inhibitors, i.e. colchicine and podophyllotoxin, not currently used in cancer therapy, were also detected as hits with scores of ~ 0.9 and ~ 0.7 in A549 and PANC-1 cells, respectively. Camptothecine and its more water-soluble semi-synthetic derivative topotecan were identified and confirmed as hits in both cell lines, with higher HTS scores obtained in the A549 cells. These compounds act as topoisomerase I inhibitors and are used to treat lung (amongst other) cancers alone and in combination with cisplatin or carboplatin [14,15].

The histone deacetylase (HDAC) inhibitor vorinostat used for the treatment of cutaneous T cell lymphoma showed strong activity in A549 cells (HTS score >0.9) and only moderate activity in PANC-1 cells (HTS score ~ 0.4) in the initial screen. Several studies for the activity of this relatively new anticancer drug, as well as of other HDAC inhibitors, against pancreatic and lung cancer cells were reported during the last decade [29–32]. Vorinostat is currently undergoing clinical trials for lung and pancreatic cancers [33,34]. The protein kinase inhibitors erlotinib and gefitinib, used in the treatment of lung and pancreatic cancers [14,35] displayed only moderate scores (0.35–0.45) in the A549 cells and were not detected as hits in PANC-1 cells.

#### PCL hits of compounds not used to treat cancers

Antiparasitic compounds represent the largest collection of drugs used to treat diseases other than cancer that were identified as hits, with HTS scores ranging between 0.3 and 1 (Fig 2 and S3 Fig). Most are benzimidazole anthelmintic drugs, e.g. parbendazole, mebendazole, albendazole, etc., which were confirmed as hits also at the lower concentration setting. The anticancer properties of mebendazole (amongst other anthelmintics) have been demonstrated in various pre-clinical studies across a number of different cancer types, including colon cancer, neuroblastoma, chemoresistant melanoma and NSCLC [36–40]. Mebendazole is currently under investigation in two clinical trials against brain tumors [41,42]. Here we show that anthelmintics are also cytotoxic to pancreatic and lung cancer cells. Other non-benzimidazole antihelmintic drugs identified in the screens, which may also be of interest for further studies as anticancer agents, include pyrvinum pamoate, hycanthone and niclosamide. Recent studies revealed the ability of pyrvinium pamoate to inhibit the growth of human pancreatic and colon cancer cells under hypoglycemic/hypoxic conditions [43,44].

Cardiac glycosides (digoxin, lanatoside C, proscilaridin A) and their aglycones (digoxigenin, digitoxigenin) exhibited strong cytotoxic effects (HTS score ≥ 0.9) in both A549 and PANC-1 cells. The activities of the cardiac glycosides remained high (score ≥ 0.9) also when tested at a lower concentration (0.83 *vs*. 10 μM) in the confirmation screen, whereas their aglycones showed slightly lower scores (see S1 and S2 Tables) and IC_50_ values between 0.2 and 0.5 μM. Cardiac glycosides are a group of naturally derived and semi-synthetic compounds with cardiotonic and antiarrhythmic properties, acting via inhibition of the cell membrane-bound Na^+^/K^+^-ATPase [45]. Some of them, for example, digoxin (S4 Fig), are used in cardiology for the treatment of congestive heart failure and atrial arrhythmias. There are several reports on the cytotoxicity of cardiac glycosides in different cancer cell lines, as well as clinical analysis and epidemiological evidence, supporting their antineoplastic effects [45–47]. Their cytotoxicity against A549 and PANC-1 was described recently as well, and IC_50_ values in the range 0.02–0.06 μM for A549 cells (48–72 h of incubation) and 0.2–0.4 μM for PANC-1 cells (48 h of incubation) were reported [48–50]. Moreover, the selectivity of cardiac glycosides towards cancer cells, compared to normal cells, has been observed [51], and different hypotheses for the mechanism of their anticancer activity have been investigated [45,47,48,52]. Bioinformatics studies suggest that the cytotoxicity of cardiac glycosides is mediated by a common mode of action different from those recognized for the known classes of anticancer drugs [46]. A limitation for their potential as anticancer drugs is their narrow therapeutic indices and low maximum tolerated doses, resulting in maximal reachable plasma concentrations below their IC_50_ values [46]. Nevertheless, some clinical evidence suggests that the doses of cardiac glycosides required for cancer treatment may be similar (or even lower) than the therapeutic plasma concentrations found in cardiac patients treated with these drugs [45]. Therefore, cardiac glycosides and their aglycones warrant further studies as anticancer drug candidates, especially for cancers with a high chemoresistance.

Several antiseptics and disinfectants were also identified as hits with pronounced activity (HTS scores > 0.9) in both cell lines. However, these hits could not be confirmed at the 10-times lower concentration setting and they are most likely characterized by an “all-or-none” effect, with their pronounced cytotoxic effects possibly resulting from nonspecific (multiple) modes of action. Similarly, high scores in both cell lines were obtained for tegaserod, auranofin and verteporfin at 10 μM doses (S5 Fig), but were not confirmed at 0.83 μM. Tegaserod is a partial 5-HT4 agonist used against constipation and for the management of irritable bowel syndrome [15]. Verteporfin is a benzoporphyrin photosensitizer clinically used for the treatment of age-related macular degeneration [15]. Its potential as photosensitizer for photodynamic therapy of locally advanced pancreatic cancer was recently demonstrated in phase I/II clinical study [53]. The gold complex auranofin is used clinically for the treatment of rheumatoid arthritis and its activity against different cancer models (including a subset of NSCLC cell lines) has been reported [54–56]. It is currently undergoing clinical trials for treatment of leukemia, as well as of some advanced solid tumors [57]. The subsequent dose-response analysis of auranofin revealed a moderate cytotoxicity with IC_50_ values of 6.65 and 2.25 μM in A549 and PANC-1 cells, respectively, with a very steep dose-response curve in A549 cells, which potentially reduces its utility as an anticancer agent. Nevertheless, its higher potency against the more chemoresistant PANC-1 cell line warrants attention, particularly given the poor success of existing treatment regimens for pancreatic cancer.

Over twenty corticosteroid drugs were detected as hits (with moderate scores 0.3–0.6), in the A549 cells. None of these compounds were identified as hits in the PANC-1 cell screens. Corticosteroids (e.g. prednisolone, methylprednisolone, dexamethasone) are used in combination chemotherapy for treatment of different type of cancers [58]. Following dose-response studies only marginal and dose-independent effects in A549 cells for the corticosteroid hits were observed.

Statins (e.g. fluvastatin, simvastatin) were identified as potential candidates for repositioning for the treatment of lung cancer with HTS scores of ca. 0.5–0.6 and IC_50_ values around 12 μM in A549 cells. A number of other compounds (e.g., haloprogin, suloctidil, terfenadine) were identified as hits with high to moderate scores in one or both cell lines, but showed high variability in the response during the screens and/or the dose-response studies, often combined with an “all-or-none” effect. Further details are provided in the Supporting Information (S1–S4 Tables, S5 Fig).

### Dose-response analysis of selected PCL hits

The cytotoxicity of representative PCL hits from the different classes was further investigated in dose-response assays to afford IC_50_ values (see Table 1, S3 and S4 Tables). The tested compounds showed diverse cytotoxicity with IC_50_ values ranging from 0.01 to 26 μM in the two cell lines. The most active compounds, with IC_50_ values <1 μM, are camptothecine, daunorubicin, doxorubicin, topotecan, cladribine, cytarabine, digoxigenin, epirubicin, gemcitabine, mebendazole and paclitaxel for the A549 cells and daunorubicin, doxorubicin, docetaxel and digitoxigenin for the PANC-1 cell line. The corticosteroid drugs, namely fludrocortisone acetate, flumethasone, hydrocortisone base and prednicarbate, exhibited only marginal, dose-independent cytotoxicity in A549 cells, failing to reach 50% inhibition of cell viability in the concentration range tested (0.8–100 μM).

**Table 1:** Cytotoxicity (expressed as IC_50_ values) of selected PCL hits in A549 and PANC-1 cells in comparison with platinum-based drugs commonly used in treatment regimens for both lung and pancreatic cancers.

PCL drug	IC_50_ [μM]^a^			
	A549		PANC-1	
Auranofin	6.65	(6.17–7.17)	2.26	(2.00–2.54)
Camptothecine(S,+)	0.15^b^	(0.11–0.19)	0.96	(0.78–1.17)
Daunorubicin.HCl	0.34^b^	(0.26–0.43)	0.26^b^	(0.18–0.37)
Doxorubicin.HCl^c^	0.26^b^	(0.21–0.35)	0.46^b^	(0.32–0.61)
Haloprogin	8.40	(7.64–9.23)	6.43	(5.23–7.91)
Topotecan	0.67^b^	(0.50–1.01)	2.54^b^	(1.16–5.54)
Vorinostat	2.32	(1.94–2.75)	10.93	(9.37–12.75)
Cladribine	0.88^b^	(0.79–0.99)	n.d.	
Cytarabine	0.43^b^	(0.32–0.59)	n.d.	
Gemcitabine	0.021^b^	(0.017–0.024)	n.d.	
Digitoxigenin	n.d.		0.18	(0.15–0.20)
Digoxigenin	0.59^b^	(0.49–0.72)	n.d.	
Fluvastatin Na	11.98	(10.83–13.25)	n.d.	
Hycanthone	n.d.		5.08	(4.22–6.11)
Mebendazole	0.65	(0.50–1.03)	n.d.	
Docetaxel	n.d.		0.019^b^	(0.008–0.032)
Paclitaxel	0.011^b^	(0.009–0.014)	n.d.	
*Cisplatin*	*5*.*03*	*(3*.*17–7*.*98)*	*22*.*48*	*(19*.*69–25*.*67)*
*Carboplatin*	*49*.*50*	*(23*.*37–104*.*9)*	*180*.*3*^*b*^	*(121*.*3–267*.*9)*
*Oxaliplatin*	*1*.*41*^*b*^	*(0*.*829–2*.*40)*	*4*.*19*^*b*^	*(3*.*28–5*.*36)*

n.d. = not determined.

^a^ 50% inhibitory concentrations with the respective 95% confidence intervals shown in brackets, obtained by the PrestoBlue assay after exposure times of 72 h; compounds were tested at a minimum of 8 concentrations, 4 replicates per concentration level.

^b^ The lower part of the curve was constrained to 0.

^c^ IC_50_ values obtained from the preliminary experiments (performed in 96-well format).

**Fig 3:**
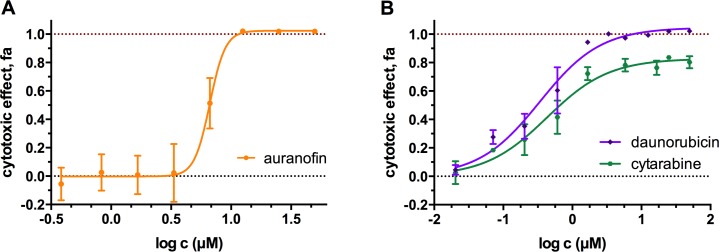
**Concentration-effect curves (means ± SD) for auranofin (A), daunorubicin and cytarabine (B) in A549 cells** (PrestoBlue fluorescence assay, 72 h exposure). Curves fitting and graphs were prepared using GraphPad Prism 6.

Some PCL hits, which showed high scores during the screens displayed steep dose-response curves (hill slope >3) with a very narrow concentration window between being inactive and completely inhibiting cell viability. Examples of compounds that behave in this way are auranofin and haloprogin in the A549 cells and β-escin, suloctidil, terfenadine and thiostreptone in the PANC-1 cells line (see S3 and S4 Tables). With β-escin, haloprogin, suloctidil, auranofin, digoxigenin, a large variation of the cell response was also observed (Fig 3A) leading to a high SDs, and consequently these compounds were not considered further. At least in some cases the variation in cell response could be attributed to the low water solubility of the compound tested. Precipitation in the wells after cells seeding was observed, e.g. for pyrvinium pamoate, terfenadine, haloprogin and thiostrepton at higher concentrations.

Not all of the active compounds with IC_50_ <1 μM were able to completely inhibit cell viability at high doses. The dose-response curves of taxanes and some of the antimetabolites plateaued at fa ≈ 0.8, i.e. cell viability reduced by 80% (see Fig 3B). This finding is in accordance with the phase-specific mode of action of taxanes and antimetabolites, which are known to be predominately cytostatic, and are only able to induce cell death in a specific part of the cell cycle [16]. In contrast, non-phase dependent cytotoxic drugs (e.g. daunorubicin) inhibit cells viability exponentially with increasing dose and are toxic to cycling cells and those in the G0 phase [16].

## Conclusions

The PCL containing 1280 drugs and drug-like molecules was screened against A549 and PANC-1 cells providing a list of compounds that could be active against highly problematic lung and pancreatic cancers. More than 100 compounds from the PCL are cytotoxic against one or both of these cells lines and, of them, 80 are drugs used to treat diseases other than cancer. The detection of anticancer drugs, which are successfully applied in the chemotherapy of lung cancer (e.g. gemcitabine, paclitaxel), as hits in A549 cells, demonstrates the applicability and credibility of the screening approach. Furthermore, some of the non-anticancer drugs that were identified as hits have already been repositioned in cancer chemotherapy and are undergoing clinical trials. Verteporfin, for example, has been shown to be a safe and effective agent for photodynamic therapy of locally advanced pancreatic cancer in phase I/II study [53]. Several promising drug candidates emanating from this study, which have the potential to be repositioned, include the antiparasitcs mebendazole, albendazole and hycanthone and the cardiac glycosides and their aglycones. Moreover, some anticancer drugs, which are currently not used in the treatment of lung and pancreatic cancers, were identified as potential candidates for these diseases (e.g. topotecan for pancreatic cancer, vorinostat for lung cancer).

It should be noted that dose-response experiments revealed that some of the very active hit compounds, e.g. auranofin, terfenadine, suloctidil and some of the antiseptics, should probably not be considered as promising anticancer drug candidates due to their very steep dose-response curves (a very narrow concentration window between no activity and complete inhibition of cell viability) and/or high variability in cell responses. Nevertheless, several new interesting lead compounds have been identified that warrant further evaluation as anticancer compounds, in particular for chemoresistant tumors.

## Materials and methods

### Compounds

The PCL was purchased from Prestwick Chemicals (Washington, DC) and comprises 1280 molecules, supplied as 10 mM stock solutions in DMSO. Compounds were stored in the dark, at– 20°C under dry air, using an automated storage system and their chemical integrity was controlled regularly by RP-HPLC, coupled to ESI-MS and CAD detector.

### Cell lines and culture conditions

A549 (human non-small cell lung adenocarcinoma) and PANC-1 (human pancreatic adenocarcinoma) cell lines were purchased from ATCC and were used between passage numbers 10 and 35. All cell culture media, buffers and reagents were obtained from Gibco Life Technologies. Cells were grown as adherent monolayer cultures in 75 cm^2^ culture flasks (TPP) without antibiotics using the following growth media, supplemented with 10% heat-inactivated fetal bovine serum (FBS, Invitrogen 10101–145): Dulbecco’s Modified Eagle medium/F-12 Nurient mixture (Ham) (DMEM/F-12 + GlutaMAX™, 31331–028) for A549 cells and Dulbecco's Modified Eagle's Medium (DMEM, high glucose, GlutaMAX™, Pyruvate; 31966–021) for PANC-1 cells. Cultures were maintained in an incubator at 37°C in a humidified atmosphere containing 5% CO_2_ and 95% air.

Cells were subcultured 2 to 3 times per week. Briefly, the cells were harvested with Trypsin 0.05%-EDTA (Life Technologies 25300062) and diluted with growth medium (1:3 to 1:5 for PANC-1 cells, 1:5 to 1:20 for A549 cells). Cells used for the assays were harvested from culture when the level of confluence was between 60% and 80%, while cell viability was > 90%.

### Screening assays development

Preliminary studies for the HTS assay development, optimization and validation for both cell lines were performed in 96-well plates (sterile, transparent, F-bottom, with lid, Cellstar 655 180), prior to upscaling to the 384-well plate format required for the screening. First, the growth kinetics and metabolic activities of the cell lines were estimated. The cells were seeded as dilution series from 2000 to 15 000 cells/well and incubated for periods from 24 to 120 h. Subsequently, dose-response studies of the cytotoxicity of doxorubicin hydrochloride were performed for different drug incubation times (i.e. 24, 48, 72 and 96 h). Drugs solutions (10 μl/well) and cell suspensions (90 μl/well) were added manually or by means of a multidrop dispenser (Thermo Scientific Multidrop Combi) into 96-well plates. In all experiments the PrestoBlue fluorescence assay for cell viability determination was used as a final read-out.

### Cell viability determination using the PrestoBlue fluorescence assay

After the respective drug exposure times, 10% final PrestoBlue (Life Technologies, Switzerland) was added to each well and the plates were returned to the incubator for 1 h. The fluorescence intensity (bottom-read) was measured using a multiwell plate reader (Tecan Infinite F500) at excitation 560/10 nm, emission 590/10 nm and a fixed gain.

### Z’-factor determination as an estimation of the assay quality

Assay quality for the different experimental setups in 96- and 384-well format was estimated by determining the Z’-factor using DOX as a positive control and DMSO as a negative control. The Z’-factor was calculated according to the formula: Z’ = 1–3(SD_pos_ + SD_neg_)/|Av_pos_−Av_neg_|, where SD and Av are the standard deviation and the average, respectively, of the fluorescence signals of the negative and positive control wells [23].

For final optimization and quality evaluation of the HTS assays, the so-called Z’-plates (in 384-well format) were prepared using half the plate as a negative control (DMSO, 0.1%) and the other half as a positive control (10 μM DOX with DMSO, 0.1%), see S6 Fig. Cells were seeded into the Z’-plates and different experimental setups (e.g. cell number, incubation time, etc.) were tested using the PrestoBlue fluorescence assay and subsequent Z’-calculation. The Z’-factor was also determined for every screen plate in all the assays, based on the controls, located in the first and last columns of the plates, in order to verify the screening assay quality and validate each plate of the screen.

### Primary HTS assay protocol

The compounds from the PCL were dispensed into sterile, basic flat-bottom, culture-treated, transparent, barcoded 384-well plates (Corning 3701), using an acoustic liquid handler Echo 550 (Labcyte Inc. Sunnyvale, CA). Each drug was added once (one well per compound), volume 30 nl, yielding a final concentration of the compound of 10 μM and final DMSO concentration of 0.1%. The first two columns of every plate were used as negative control (no PCL drug added) and filled with an equivalent volume of DMSO (30 nl/well) while the last two columns were filled with doxorubicin (10 μM with DMSO, 0.1%) for the positive control (see Fig 1 for the plate layout). Cells were harvested by tripsinization and seeded in complete growth medium into the plates in volumes of 30 μl/well using a multi-drop dispenser (Thermo Scientific Multidrop Combi) at medium speed. A seeding density of 2000 cells/well was used for both A549 and PANC-1 cells to ensure exponential growth of untreated controls throughout the experiment and a satisfactory quality of the assay (Z’ >0.5). After a post-plating incubation step at room temperature for 20 min, which ensures a more homogeneous repartition of the cells at the bottom of the wells, plates were transferred to an incubator (37°C, 5% CO_2,_ saturated moist atmosphere) for 50 h (A549 cells) or 72 h (PANC-1 cells). The PrestoBlue fluorescence assay for cell viability determination was used as a final read-out for the screen as described above. Data was normalized according to the fluorescence signals of control wells located in the first two and last two columns of every plate and presented as HTS scores, where a score of 0 was assigned to the average fluorescence intensity of the negative control wells and 1 to that of the positive control wells. PCL compounds with HTS scores higher than the average of the negative controls + 3 x SD were classified as ‘Hit compounds’. An in-house Laboratory Information Management System (LIMS) was used for basic data processing, management, visualization and statistical hit validation.

Screening of PCL in each of the cell lines was repeated three times (each time in duplicate), ensuring in total six wells per compound and primary hit scores were calculated as average ± SD.

### Confirmation screening

The hit compounds from the primary screening assay were dispensed (cherry picked) into 384-well plates using the acoustic liquid handler Echo 550 (Labcyte Inc. Sunnyvale, CA). Compounds were added in volumes of 30 nl or 2.5 nl (for hits with scores > 0.65 in the primary screen) to give final concentrations of 10 μM or 0.83 μM, respectively. All conditions were assayed in duplicate. The plate layout was redesigned to accommodate the lower number of samples and avoid the edge effect (no controls or compounds in the edge wells). Cell seeding, incubation, cell viability determination and data evaluation were as described for the primary screening.

### Hits dose response studies and IC_50_ determination

Selected PCL compounds were further tested for their cytotoxicity in a dose-response manner in 384-well plates using the PrestoBlue fluorescence assay. Compound dilution series (dilution factors 1:2 or 1:3) were generated using an Echo dispenser. Compounds were tested at a minimum of 8 concentrations, with 4 replicates per concentration (i.e. 2 wells/plate, every plate in duplicate). Cells were seeded in volumes of 30 μl/well (using a multi-drop dispenser) at seeding density of 2000 cells/well for PANC-1 cells and 1250 cells/well for A549 cells. Incubation time was set to 72 h for both cell lines in order to simplify comparison. Dose-response curves and IC_50_ determination were generated by using the in-house developed software within LIMS based on four-parameters nonlinear regression (variable slope) model with a fixed bottom (0 effect), when necessary.

## Supporting information

S1 Fig Age-standardized ten-year net survival trends, adults (aged 15–99), selected cancers, England and Wales, 1971–2011 (according to: Cancer Research UK: Cancer survival for common cancers. Available from: http://www.cancerresearchuk.org/health-professional/cancer-statistics/survival/common-cancers-compared#heading-Three, accessed on 28.04.2016).
(TIFF)

S2 Fig Chemical structure of doxorubicin (R = CH_2_OH) and daunorubicin (R = CH_3_). (TIF)

S3 Fig Antiparasitic agents identified as hits in A549 and/or PANC-1 cells, plot based on their mean scores from the primary screens. Hits identified in only one of the cell lines are plotted and their score on the other is taken as zero.
(TIFF)

S4 Fig Structure of digoxin; the non-sugar part (aglycone, colored in red) is named digoxigenin. (TIF)

S5 Fig Miscellaneous hits identified in A549 and/or PANC-1 cells, plot based on their mean scores from the primary screens. Hits identified in only one of the cell lines are plotted and their score on the other is taken as zero.
(TIFF)

S6 Fig Schematic of a typical 384-well Z’-plate: Left: A general view of the PrestoBlue readout; Right: The corresponding data evaluation, visualization and Z’-calculation. The plate was seeded with 1200 A549 cells/well with first half containing 0.1% DMSO and the second half 10 μM Doxorubicin and final DMSO concentration 0.1%. Incubation time = 72 h.
(TIFF)

S1 Table Hits identified after screening 1280 PCL compounds against PANC-1 cells (exposure time of 72 h). Scores from the primary screen are given as mean and SD from 3 independent experiments, performed in duplicate (6 wells/compound). Data for the confirmation screen is based on one experiment, performed in duplicate (2 wells/drug). Compounds annotation and classification is based on the Anatomical Therapeutic Chemical (ATC) Classification System, the PCL annotation file and Martindale.
* SD only from one independent experiment, 2 wells/drug; ** SD only from two independent experiments, 3–4 wells/drug; blank wells mean that the compound was not tested at the particular condition, while ‘-‘ means that it was not confirmed as hit in the particular assay; color-code is based on the herein accepted pharmacological classification.
(XLSX)

S2 Table Hits identified after screening 1280 PCL compounds against A549 cells (exposure time of 50 h). Scores from the primary screen are given as mean and SD from 3 independent experiments, performed in duplicate (6 wells/compound). Data for the confirmation screen is based on two experiments, performed in duplicate (4 wells/drug); Compounds annotation and classification is based on the Anatomical Therapeutic Chemical (ATC) Classification System, the PCL annotation file and Martindale.
* SD only from one independent experiment, 2 wells/drug; ** SD only from two independent experiments, 3–4 wells/drug; blank wells mean that the compound was not tested at the particular condition, while ‘-‘ means that it was not confirmed as hit in the particular assay; coloring is based on the herein accepted pharmacological classification.
(XLSX)

S3 Table In vitro cytotoxicity of the investigated PCL hits in A549 cells in comparison to the platinum drugs commonly used in treatment regimens of lung cancer. ^a^ 50% inhibitory concentrations with the respective 95% confidence intervals shown in brackets, obtained by the presto blue assay after exposure times of 72 h; compounds were tested at minimum 8 concentrations, 4 replicates per concentration level and in 384-well format.
^b^ The bottom of the curve was constrained to zero.
^c^ Precipitation observed after cell seeding at high concentrations.
(DOCX)

S4 Table In vitro cytotoxicity of the investigated PCL hits in PANC-1 cells in comparison to platinum drugs, commonly used in treatment regimens of pancreatic cancer. ^a^ 50% inhibitory concentrations with the respective 95% confidence intervals shown in brackets, obtained by the presto blue assay after exposure times of 72 h; compounds were tested at minimum 8 concentrations, 4 replicates per concentration level and in 384-well format.
^b^ The top had to be constrained to 1 (as e.g. the curve cannot reach plateau at the highest concentrations tested).
^c^ The bottom of the curve was constrained to 0.
^d^ Precipitation observed after cell seeding at high concentrations.
(DOCX)
