# Supplementary figures and images for: Repositioning approved drugs for the treatment of problematic cancers using a screening approach
Source: PLoS One. 2017 Feb 6;12(2):e0171052. doi: 10.1371/journal.pone.0171052 (PMC5293254; doi:10.1371/journal.pone.0171052)

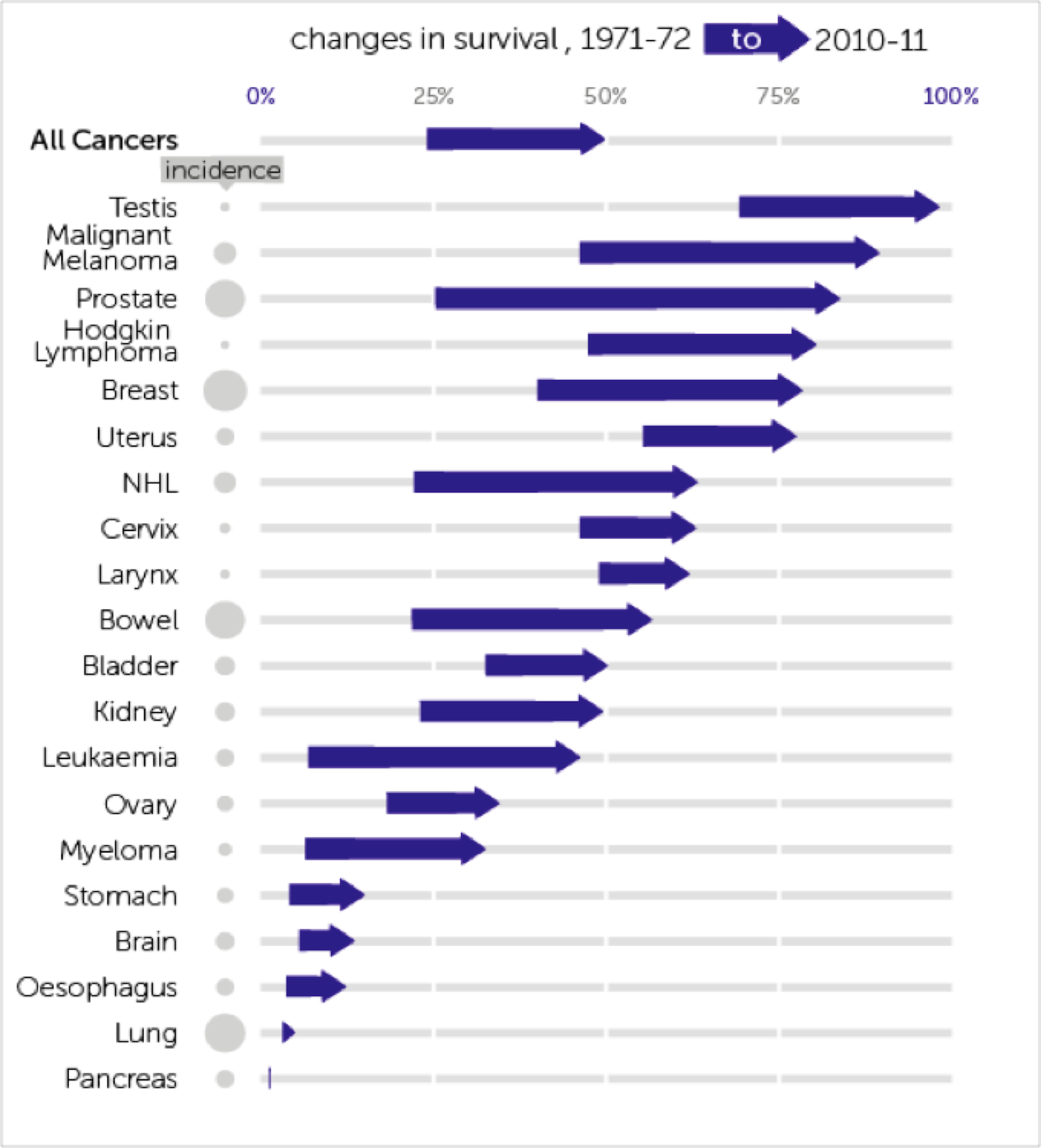

Supplement: S1 Fig — Available from: http://www.cancerresearchuk.org/health-professional/cancer-statistics/survival/common-cancers-compared#heading-Three, accessed on 28.04.2016). (TIFF) [file pone.0171052.s001.tiff]

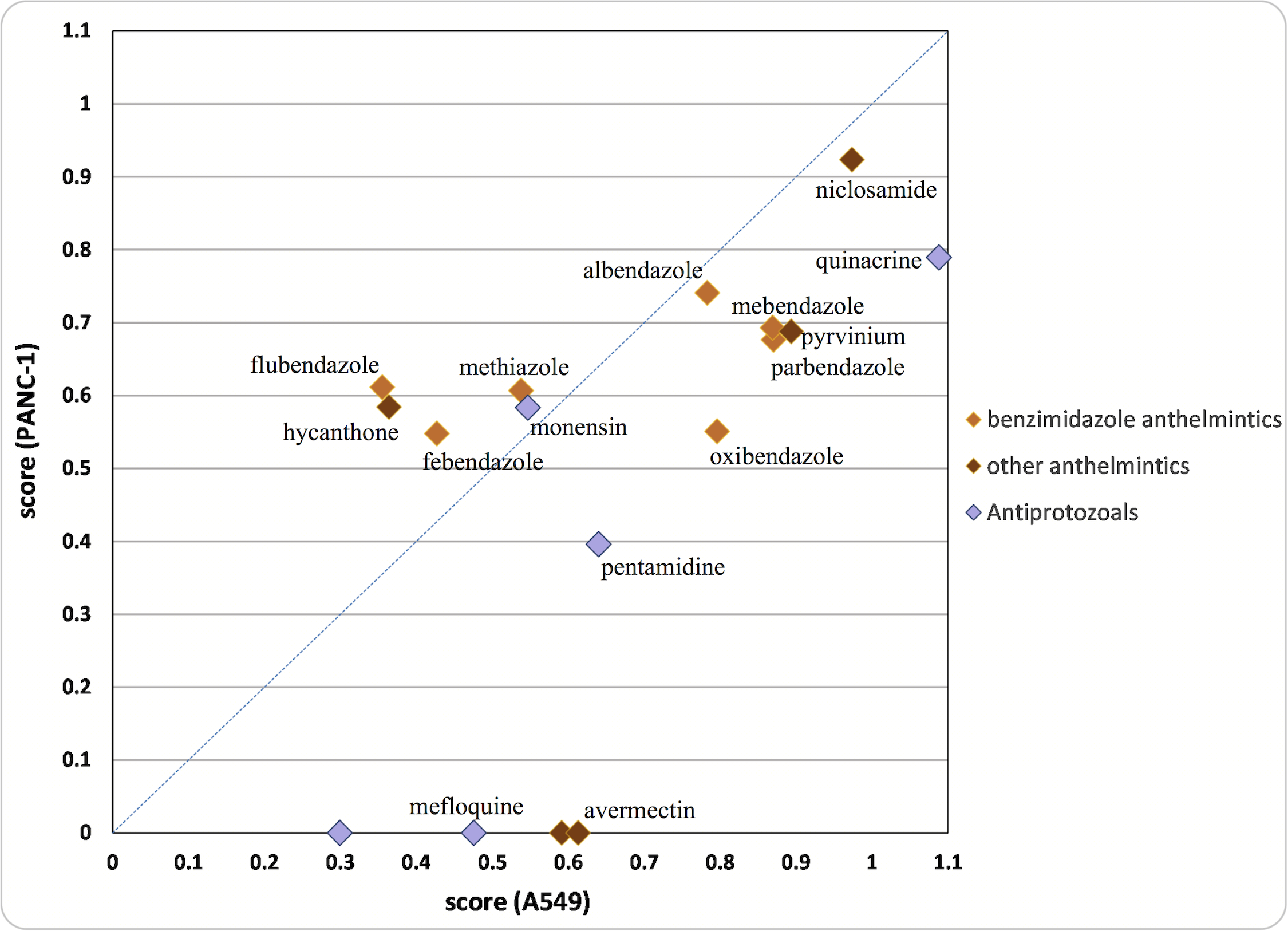

Supplement: S3 Fig — Hits identified in only one of the cell lines are plotted and their score on the other is taken as zero. (TIFF) [file pone.0171052.s003.tiff]

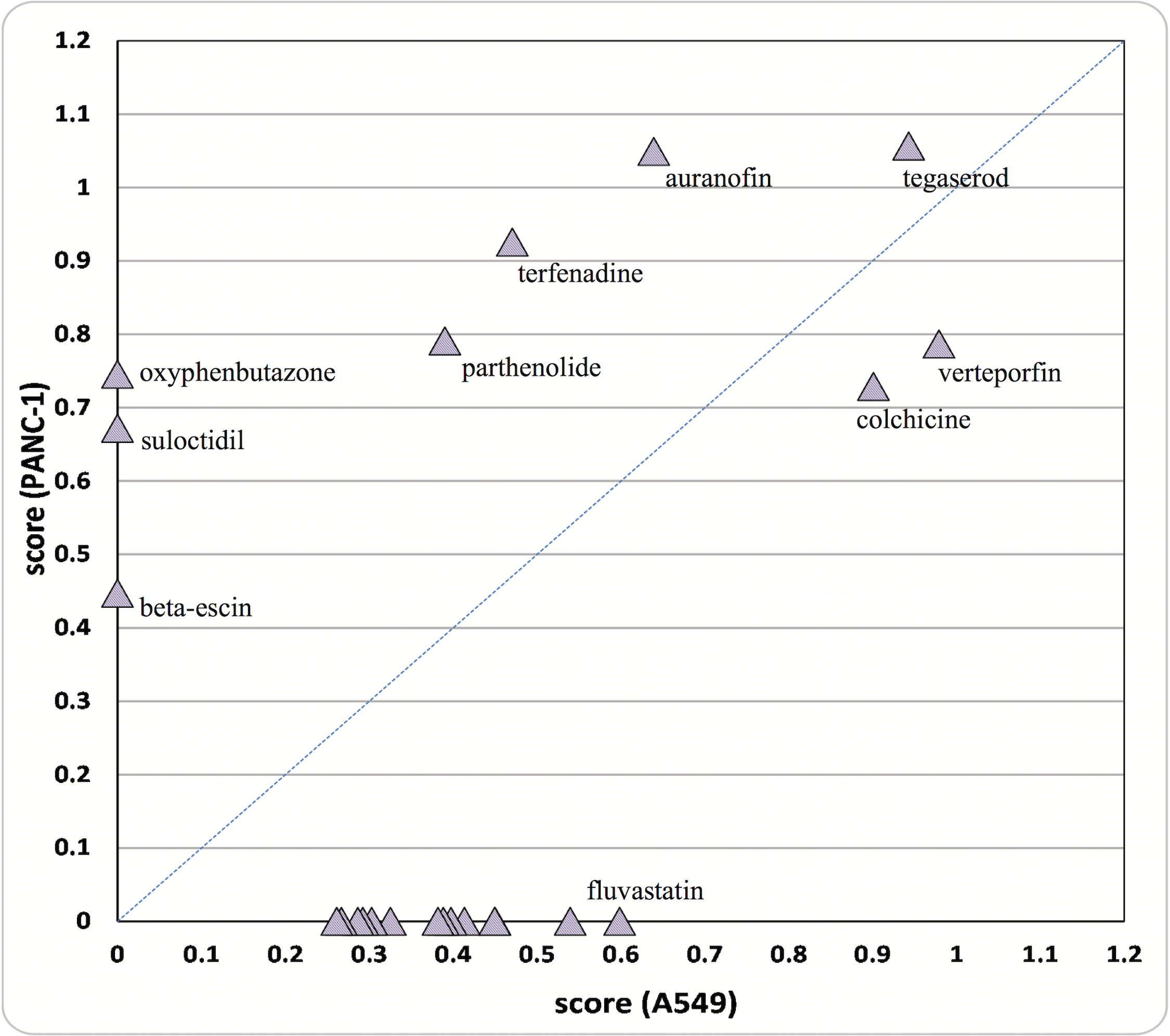

Supplement: S5 Fig — Hits identified in only one of the cell lines are plotted and their score on the other is taken as zero. (TIFF) [file pone.0171052.s005.tiff]

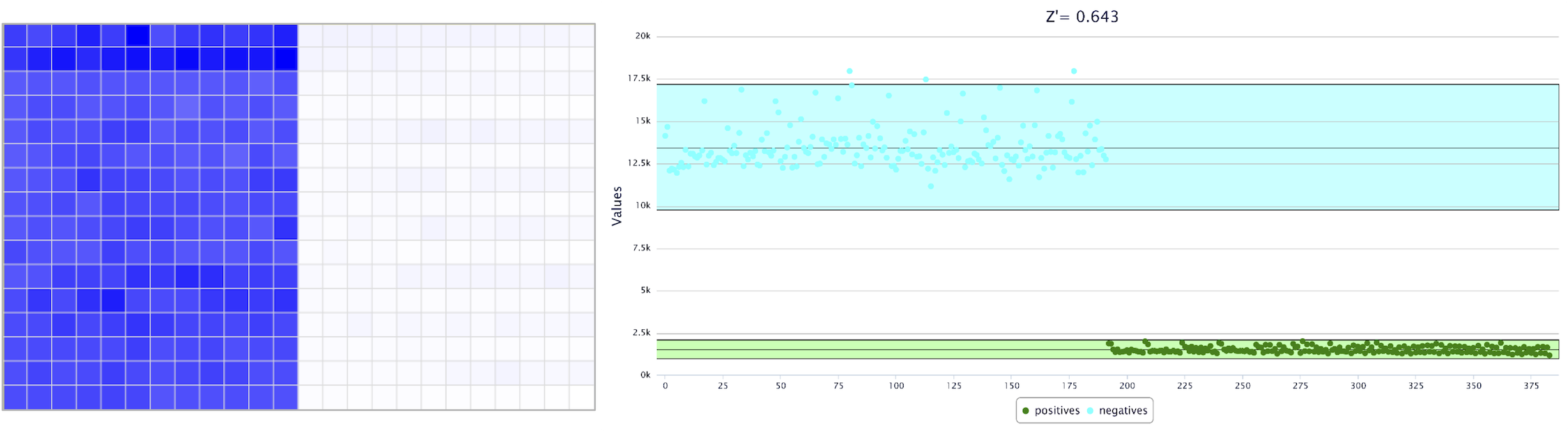

Supplement: S6 Fig — The plate was seeded with 1200 A549 cells/well with first half containing 0.1% DMSO and the second half 10 μM Doxorubicin and final DMSO concentration 0.1%. Incubation time = 72 h. (TIFF) [file pone.0171052.s006.tiff]
