# Supplementary material for: Repositioning approved drugs for the treatment of problematic cancers using a screening approach
Source: PLoS One. 2017 Feb 6;12(2):e0171052. doi: 10.1371/journal.pone.0171052 (PMC5293254; doi:10.1371/journal.pone.0171052)
Supplement: S3 Table — a 50% inhibitory concentrations with the respective 95% confidence intervals shown in brackets, obtained by the presto blue assay after exposure times of 72 h; compounds were tested at minimum 8 concentrations, 4 replicates per concentration level and in 384-well format. b The bottom of the curve was constrained to zero. c Precipitation observed after cell seeding at high concentrations. (DOCX) [file pone.0171052.s009.docx]

**Table S3.** In vitro cytotoxicity of the investigated PCL hits in A549 cells in comparison to the platinum drugs commonly used in treatment regimens of lung cancer.

| **Name** | **Top Asymptote** | **Bottom Asymptote** | **Hill Slope** | **R^2^** | **IC_50_^a^** | **95% CI** |
| --- | --- | --- | --- | --- | --- | --- |
| *Cisplatin* | *1.065* | *-0.088* | *1.110* | *0.95* | *5.029* | *(3.170-7.977)* |
| *Carboplatin* | *1.000* | *-0.048* | *1.040* | *0.94* | *49.50* | *(23.37-104.9)* |
| *Oxaliplatin^b^* | *0.860* | *0.000* | *2.018* | *0.95* | *1.410* | *(0.829-2.397)* |
| Auranofin | 1.024 | -0.002 | 6.709 | 0.95 | 6.65 | (6.166-7.167) |
| Azacytidine-5 | 0.932 | -0.071 | 1.148 | 0.98 | 17.93 | (13.16-24.43) |
| Camptothecine (S,+)*^b^* | 1.043 | 0.000 | 0.804 | 0.95 | 0.145 | (0.111-0.191) |
| Cladribine*^b^* | 0.896 | 0.000 | 1.612 | 0.97 | 0.883 | (0.790-0.986) |
| Cytarabine*^b^* | 0.828 | 0.000 | 0.970 | 0.94 | 0.430 | (0.316-0.586) |
| Daunorubicin.HCl*^b^* | 1.047 | 0.000 | 0.985 | 0.96 | 0.336 | (0.265-0.427) |
| Deferoxamine mesylate | 0.717 | -0.001 | 2.166 | 0.95 | 6.367 | (5.364-7.558) |
| Digoxigenin*^b^* | 0.969 | 0.000 | 2.389 | 0.95 | 0.594 | (0.490-0.720) |
| Epirubicin.HCl*^b^* | 1.024 | 0.000 | 0.876 | 0.93 | 0.234 | (0.183-0.297) |
| Erlotinib | 0.738 | 0.205 | 2.348 | 0.97 | 8.806 | (7.602-10.20) |
| Fluvastatin Na | 0.929 | 0.022 | 2.544 | 0.98 | 11.98 | (10.83-13.25) |
| Gemcitabine*^b^* | 0.780 | 0.000 | 1.872 | 0.94 | 0.021 | (0.017-0.024) |
| Haloprogin^c^ | 1.026 | 0.005 | 3.660 | 0.98 | 8.400 | (7.645-9.230) |
| Mebendazole | 0.799 | -0.034 | 2.466 | 0.94 | 0.650 | (0.495-1.030) |
| Paclitaxel*^b^* | 0.805 | 0.000 | 1.301 | 0.86 | 0.011 | (0.009-0.014) |
| Topotecan*^b^* | 1.008 | 0.000 | 0.941 | 0.90 | 0.669 | (0.498-1.009) |
| Vorinostat | 1.005 | 0.028 | 1.606 | 0.96 | 2.316 | (1.936-2.747) |

^a^ 50% inhibitory concentrations with the respective 95% confidence intervals shown in brackets, obtained by the presto blue assay after exposure times of 72 h; compounds were tested at minimum 8 concentrations, 4 replicates per concentration level and in 384-well format.

^b^ The bottom of the curve was constrained to zero.

^c^ Precipitation observed after cell seeding at high concentrations.
