# Supplementary material for: Repositioning approved drugs for the treatment of problematic cancers using a screening approach
Source: PLoS One. 2017 Feb 6;12(2):e0171052. doi: 10.1371/journal.pone.0171052 (PMC5293254; doi:10.1371/journal.pone.0171052)
Supplement: S4 Table — a 50% inhibitory concentrations with the respective 95% confidence intervals shown in brackets, obtained by the presto blue assay after exposure times of 72 h; compounds were tested at minimum 8 concentrations, 4 replicates per concentration level and in 384-well format. b The top had to be constrained to 1 (as e.g. the curve cannot reach plateau at the highest concentrations tested). c The bottom of the curve was constrained to 0. d Precipitation observed after cell seeding at high concentrations. (DOCX) [file pone.0171052.s010.docx]

**Table S4.** In vitro cytotoxicity of the investigated PCL hits in PANC-1 cells in comparison to platinum drugs, commonly used in treatment regimens of pancreatic cancer.

| **Name** | **Top Asymptote** | **Bottom Asymptote** | **Hill Slope** | **R^2^** | **IC_50_^a^** | **95% CI** |
| --- | --- | --- | --- | --- | --- | --- |
| *Cisplatin* | *1.026* | *0.242* | *2.791* | *0.97* | *22.48* | *(19.69-25.67)* |
| *Carboplatin^b,c^* | *1.000* | *0.000* | *0.705* | *0.79* | *180.3* | *(121.3-267.9)* |
| *Oxaliplatin^b,c^* | *1.000* | *0.000* | *0.500* | *0.87* | *4.193* | *(3.279-5.361)* |
| Aminacrine*^b,c^* | 1.000 | 0.000 | 1.595 | 0.94 | 1.924 | (1.535-2.411) |
| Auranofin | 1.073 | -0.020 | 2.559 | 0.98 | 2.255 | (2.003-2.538) |
| Beta-Escin | 1.060 | 0.036 | 4.286 | 0.90 | 26.82 | (22.43-32.07) |
| Camptothecine (S,+) | 0.830 | 0.262 | 1.411 | 0.98 | 0.955 | (0.778-1.173) |
| Ciclopirox ethanolamine^c^ | 0.748 | 0.000 | 2.118 | 0.91 | 1.439 | (1.185-1.748) |
| Daunorubicin.HCl^c^ | 1.128 | 0.000 | 0.660 | 0.95 | 0.256 | (0.176-0.373) |
| Digitoxigenin | 0.908 | -0.066 | 2.158 | 0.98 | 0.175 | (0.154-0.198) |
| Haloprogin^d^ | 1.063 | -0.072 | 2.127 | 0.97 | 6.433 | (5.234-7.908) |
| Hycanthone | 1.060 | -0.021 | 1.715 | 0.97 | 5.079 | (4.223-6.108) |
| Pyrvinium pamoate^d^ | 0.964 | -0.219 | 2.125 | 0.98 | 3.740 | (3.099-4.513) |
| Suloctidil | 1.056 | 0.031 | 5.853 | 0.98 | 8.678 | (7.959-9.462) |
| Terfenadine^d^ | 1.062 | 0.083 | 5.172 | 0.97 | 16.62 | (14.80-18.66) |
| Thiostrepton^d^ | 1.003 | 0.022 | 4.487 | 0.98 | 4.118 | (2.948-5.753) |
| Topotecan^c^ | 0.987 | 0.000 | 0.987 | 0.91 | 2.536 | (1.162-5.537) |
| Vorinostat | 0.836 | -0.066 | 1.823 | 0.98 | 10.93 | (9.361-12.75) |

^a^ 50% inhibitory concentrations with the respective 95% confidence intervals shown in brackets, obtained by the presto blue assay after exposure times of 72 h; compounds were tested at minimum 8 concentrations, 4 replicates per concentration level and in 384-well format.

^b^ The top had to be constrained to 1 (as e.g. the curve cannot reach plateau at the highest concentrations tested).

^c^ The bottom of the curve was constrained to 0.

^d^ Precipitation observed after cell seeding at high concentrations.
